# Predictors of severe leptospirosis: a multicentre observational study from Central Malaysia

**DOI:** 10.1186/s12879-021-06766-5

**Published:** 2021-10-19

**Authors:** Noraini Philip, Leslie Thian Lung Than, Anim Md Shah, Muhamad Yazli Yuhana, Zamberi Sekawi, Vasantha Kumari Neela

**Affiliations:** 1 Department of Medical Microbiology, Faculty of Medicine and Health Sciences, Universiti Putra Malaysia, Serdang, Selangor Malaysia; 2 Department of Medicine, Faculty of Medicine and Health Sciences, Universiti Putra Malaysia, Serdang, Selangor Malaysia; 3 Infectious Diseases Unit, Internal Medicine Department, Universiti Teknologi MARA, Sungai Buloh, 47000 Selangor, Malaysia

**Keywords:** Leptospirosis, *Leptospira*, Mild, Severe, Predictors

## Abstract

**Background:**

Leptospirosis is a re-emerging disease with vast clinical presentations, that ranges from subclinical or mild to severe and fatal outcomes. Leptospirosis can be managed well if diagnosed earlier, however, similar clinical presentations by several other febrile illnesses or co-infections, and laboratory diagnostic challenges due to the biphasic nature of the illness, often result in mis- or underdiagnosis, thereby lead to severe illness. Identification of clinical predictors for the severe form of the disease plays a crucial role in reducing disease complication and mortality. Therefore, we aimed to determine the clinical predictors associated with severe illness among leptospirosis patients from Central Malaysia through a prospective multicenter observational study.

**Methods:**

A prospective multicenter observational study was performed on patients admitted for clinically suspected leptospirosis. Three hospitals namely Hospital Serdang, Hospital Tengku Ampuan Rahimah and Hospital Teluk Intan were included in the study. Among a total of 165 clinically suspected leptospirosis patients, 83 confirmed cases were investigated for clinical predictors for severe illness. Qualitative variables were performed using χ2 and the relationship between mild and severe cases was evaluated using logistic regression. Multivariable logistic regression was used to predict the independent variable for severity.

**Results:**

Among the 83 patients, 50 showed mild disease and 33 developed severe illness. The mean age of the patients was 41.92 ± 17.99 and most were males (n = 54, 65.06%). We identified mechanical ventilation, acute kidney injury, septic shock, creatinine level of > 1.13 mg/dL, urea > 7 mmol/L, alanine aminotransferase > 50 IU, aspartate aminotransferase > 50 IU, and platelet < 150 × 10^9^/L as factors associated with severe illness. Acute kidney injury, alanine aminotransferase > 50 IU and platelet < 150 × 10^9^/L were defined as the independent factors for severity.

**Conclusions:**

Lungs, liver and kidney involvement and septic shock were found as the prognostic factors for severe leptospirosis. Acute kidney injury, high level of alanine aminotransferase and low level of platelets were found to be independent predictors of severity.

## Background

Leptospirosis is a globally distributed zoonotic disease caused by the pathogenic species of *Leptospira*, a genus of spirochete bacteria. Although this disease has a worldwide distribution, it is more common in the tropics due to its favorable transmission condition. In Malaysia, leptospirosis is an endemic disease. Occasional outbreaks have been reported and are mainly associated with forest and water activities, flood and crowded living environment [1–5].

The clinical presentation of leptospirosis varies from mild to severe, life-threatening and fatal outcomes. The symptoms are diverse, generally include fever and often shares presentations with other infectious diseases, such as malaria, dengue, and influenza. About 90% of leptospirosis is a subclinical or self-limited febrile illness, while the severe form is presented by sepsis and multiple organ failures [6]. The most severe form of leptospirosis is Weil’s disease, seen in 5–15% of infected cases and typically involves multiple organs damages accompanied with jaundice, acute renal failure and hemorrhage and has a fatality rate of more than 10% [7, 8]. However, leptospirosis cases with lung involvement presenting with acute respiratory distress syndrome (ARDS) or severe pulmonary hemorrhage syndrome (SPHS) have the highest mortality rates of over 70% [7]. The presentation of leptospirosis appears to be distinct in different geographical areas worldwide [8]. This is particularly true as different geographical location may have different prevailing *Leptospira* species and serovars, socio-economy and environmental conditions. The variations of intrinsic virulence among serovars and species have been asserted to partially explain the disease severity of mild and severe forms of leptospirosis [9]. These differences may also lead to specific predictors of severe disease and mortality.

The two extreme manifestations of leptospirosis which are mild/subclinical and severe illness with mortality caution that the disease should not be taken lightly, and constant monitoring of patients is essential. For constant monitoring, it is important to determine the prognostic markers, but it is still not clear what factors that able to predict the transition from mild to severe/fatal illness. Several independent prognostic factors for fatal leptospirosis have been reported across the globe and these include older age, oliguria, hyperkalaemia, abnormal serum creatinine level, ARDS, pulmonary haemorrhage, elevated bilirubin level, hypotension, arrhythmia and altered mental status [8, 10, 11].

Earlier studies from Malaysia identified elevated levels of cytokines such as IL-6, IL-17A and IL-22 as potential prognostic biomarkers with IL-17A as the independent predictor for leptospirosis associated fatalities [12]. Another study conducted in a suburban area in Malaysia identified age above 70, clinical presentations suggestive of organ dysfunction and intensive care requirement as predictors for fatalities [13]. Fish Low et al. (2020) reported hypocalcemia (calcium < 2.10 mmol/L), hypochloremia (chloride < 98 mmol/L), and eosinopenia (absolute eosinophil count < 0.040 × 10^9^/L) as clinical predictors for laboratory-confirmed leptospirosis when compared with clinically suspected leptospirosis cases [14]. Analysis of a set of data for clinically diagnosed leptospirosis cases in a tertiary care hospital in Malaysia showed gastrointestinal tract (GIT) presentation as one of the predictors for severe illness and the study also found that GIT symptoms are significantly associated with age (20–40 years), poor sanitation and crowded living environment of the patients [15].

In the present investigation, we performed a multicentered observational study to identify factors predictive of severe leptospirosis. Determining the predictors at the early stage of the disease could greatly reduce the severe illness development and thereby mortality.

## Methods

### Study design

The study was conducted from January 2016 to December 2017 in three tertiary hospitals from two states in Central Malaysia: Hospital Serdang and Hospital Tengku Ampuan Rahimah (HTAR) in the state of Selangor and Hospital Teluk Intan in the state of Perak. A prospective multicenter observational study was performed on patients with clinically suspected leptospirosis. Among the 165 suspected patients, 92 were confirmed leptospirosis [1, 19]. The laboratory confirmation of these patients is defined as positive by polymerase chain reaction (PCR) or microscopic agglutination test (MAT) (first sample > 1:400; seroconversion or fourfold increase in the paired samples) [16]. The genes used to detect the presence of leptospires in blood and serum were *lipL32* and 16S rDNA [17, 18]. The samples positive by 16S rDNA were subjected to sequencing (MyTACG Bioscience Enterprise, Malaysia) to identify the infecting *Leptospira* species [1]. The serovars used for MAT assay comprised of Australis, Autumnalis, Batavia, Canicola, Celledoni, Grippotyphosa, Hardjoprajitno, Icterohaemorrhagiae, Javanica, Pyrogenes, Tarrasovi, Djasiman, Patoc, Pomona (international serovars were obtained from World Health Organization (WHO)) and IMR LEP 1; saprophyte, IMR LEP 115; saprophyte, IMR LEP 175; saprophyte, IMR LEP 803/11-Copenhageni, IMR LEP 27-Hardjobovis, IMR LEP 22-Lai (local serovars were obtained from the Institute for Medical Research (IMR), Federal Territory of Kuala Lumpur, Malaysia [19, 20]. Among the 92 confirmed leptospirosis, a total of 83 patients with complete clinical admission data were recruited for classification of mild and severe (severe included dead patients) categories.

### Case definitions

Mild illness is characterized by fever, headache, myalgia, conjunctival congestion, mild cough, lymphadenopathy, rash, anorexia, nausea and vomiting [21], while severe illness is defined as hospitalization plus jaundice, acute kidney injury, and or pulmonary involvement [1, 22]. The classification of mild and severe patients was based on the clinical admission data.

### Statistical analysis

All data such as the socio-demography of the patients, clinical presentation and selected blood profile were entered and recorded in Excel sheet. Epi info version 7 was used for analysis. Qualitative variables were compared through χ^2^ test. The relationship between mild and severe cases was evaluated using logistic regression, using severe as the dependent or outcome variable. Variables which had significant finding in univariate analysis for the severe cases were further analysed by multivariable logistic regression test to predict the independent variable for severity. Only p-value less than 0.05 with a corresponding 95% confidence interval (CI) of more than one was taken as a significant result.

## Results

### Descriptive analysis

Overall, among the 83 positive patients, 50 were identified as mild and 33 as severe cases. A total of 57 patients were confirmed by 16S rDNA and the infecting species were identified as *L. interrogans* (n = 40; 48.19%), *L. kirschneri* (n = 16; 19.28%) and *L. wolffii* (n = 1; 1.20%). The socio-demography, clinical presentations and laboratory findings of all confirmed patients are shown in Table 1. The 83 patients comprised of 54 (65.06%) males and 29 (34.94%) females with a mean age of 41.92 years. Twenty patients had acute kidney injury (AKI). Of the 83 confirmed leptospirosis patients, 11 (13.25%) died and 72 (86.75%) recovered. Among the 72 patients who recovered, 22 developed severe illness based on the definition given above (case definition in the methods section).

**Table 1:** Socio-demographic, clinical presentation and laboratory findings

Socio-demographic, clinical presentations and laboratory findings	Mean ± SD or n (%)
Socio-demographic	
Gender	
Male	54 (65.06%)
Female	29 (34.94%)
Age	41.92 ± 17.99
> 60	20(24.10%)
< 60	63(75.90%)
Clinical presentations	
Fever	81 (97.59%)
Headache	34 (40.96%)
GIT involvement	53 (63.86%)
Myalgia	27 (32.53%)
Mechanical ventilation	6 (7.23%)
AKI	20 (24.10%)
Septic shock	9 (10.84%)
Laboratory findings	
Creatinine > 1.13 mg/dL	35 (42.17%)
Urea > 7 mmol/L	33 (39.76%)
Bilirubin > 32.49 μmol/L	20 (24.39%)
ALT > 50 IU	35 (42.17%)
AST > 50 IU	45 (55.56%)
HCT < 35%	38 (47.50%)
Platelet < 150 × 10^9^/L	30 (36.14%)
HB < 11.5 g/dL	35 (42.17%)
WBC < 11 × 10^9^/L	34 (40.96%)
Infecting species	
*Leptospira interrogans*	40 (48.19%)
*Leptospira kirschneri*	16 (19.28%)
*Leptospira wolffii*	1 (1.20%)

*SD* standard deviation, *n* total number

### Univariate analysis

Univariate analysis identified requirement of mechanical ventilation, AKI, septic shock, creatinine level of > 1.13 mg/dL, urea > 7 mmol/L, ALT > 50 IU, AST > 50 IU, and platelet < 150 × 10^9^/L as significant association with severe diseases (Table 2).

**Table 2:** Univariate analysis between severe and mild infections

Variables	Severe cases (n = 33, 39.8%)	Mild cases (n = 50, 60.2%)	Odds ratio	95% CI	χ^2^ test	Corrected X^2^ tailed p p < 0.05
Gender M:F	(70%, M)	(62%, M)	1.4	0.6–3.6	0.2	0.6
Older age > 60 years	27%	22%	1.3	0.4–4.1	0.08	0.7
Fever	97%	98%	0.7	0.008–52.8	UN	1^#^
Headache	30%	48%	0.5	0.2–1.2	1.9	0.2
GIT involvement	55%	70%	0.5	0.2–1.3	1.4	0.2
Myalgia	36%	30%	1.3	0.5–3.4	0.1	0.7
Mechanical ventilation	18%	0%	UN	UN	UN	0.0029**^#^
AKI	48%	8%	10.8	2.9–49.2	15.7	0.00007**
Septic shock	21%	4%	6.5	1.1–66.6	4.4	0.035**
Creatinine > 1.13 mg/dL	70%	24%	7.3	2.7–19.5	15.2	0.00009**
Urea > 7 mmol/L	67%	22%	7.1	2.6–19.0	14.7	0.0001**
Bilirubin > 32.49 μmol	33%	18%	2.2	0.7–7.1	1.7	0.2
ALT > 50 IU	58%	32%	2.9	1.2–7.2	4.3	0.037**
AST > 50 IU	79%	40%	5.7	2.1–15.6	10.6	0.001**
HCT < 35%	56%	42%	1.8	0.7–4.4	1.1	0.3
Platelet < 150 × 10^9^/L	58%	22%	4.8	1.8–12.6	9.4	0.002**
HB < 11.5 g/dL	48%	38%	1.5	0.6–3.7	0.5	0.5
WBC < 11 × 10^9^/L	42%	40%	1.1	0.5–2.7	0.0	1
*Leptospira interrogans*	68%	71%	0.9	0.2–3.3	0.0	1
*Leptospira kirschneri*	32%	26%	1.4	0.3–5.1	0.04	0.8

*UN* undefined

^#^p-value based on Fisher-exact test

**Significant value

### Multivariable analysis

Multivariable analysis on variables that showed significant association with severe cases, performed through the multivariable logistic regression analysis found that AKI with odds ratio (OR) 10.4 (1.1–100.6), ALT > 50 IU with OR 8.1 (1.2–52.5) and platelet < 150 × 10^9^/L with OR 7.3 (1.5–33.9) were the independent risk factors associated with severity (Table 3).

**Table 3:** Multivariable logistic regression using severe as the dependent variable for the independent predictor of severity in leptospirosis

Variables	Odds Ratio	95% CI	Coefficient	S.E	Z-statistic	p-value
AKI	10.4	1.1–100.6	2.3	1.2	2.0	0.04**
Mechanical ventilation	401,432.1	0.0–1.0 × 10^12^	12.9	263	0.05	0.96
Septic shock	0.2	0.01–4.3	− 1.4	1.5	− 0.9	0.3
ALT > 50 IU	8.1	1.2–52.5	2.1	0.9	2.1	0.03**
AST > 50 IU	1.9	0.3–10.8	0.7	0.9	0.7	0.5
Creatinine > 1.13 mg/dL	1.8	0.2–13.6	0.6	1.0	0.5	0.6
Urea > 7 mmol/L	3.8	0.6–25.3	1.3	0.9	1.3	0.2
Platelet < 150 × 10^9^/L	7.3	1.6–33.9	1.9	0.8	2.6	0.01**
CONSTANT	*	*	− 16.3	263.5	− 0.06	0.95

**Significant value

## Discussion

The clinical manifestation and presentation of leptospirosis are broad and only specific when it becomes severe. This presents challenges to clinicians not only to make a correct diagnosis but also to give the best management and treatment to the patients to prevent progressing to severe disease and mortality. Several studies have outlined the prognostic factors associated with severe and fatal leptospirosis, however, the predictors could be different between geographical location depending on the socio-demography, type of occupation, prevailing *Leptospira* species, serovars and strains [8, 23].

In the present study, most leptospirosis patients were male, and this might be due to exposure-related bias where male are more involved with outdoor activities and may have occupational-related risk exposure. Patients were presented with the typical manifestation of leptospirosis such as fever, headache, myalgia and gastrointestinal involvement such as nausea and abdominal pain. Age above 70 years together with organ dysfunction has been reported as a predictor for fatality in an earlier study from Malaysia [13], however, we did not find any significant association with age and severity of the illness in this present study. It is undoubtedly evident that older age worsens the disease course, and the reason it was not significant for severity in our study, could be due to the low number of patients aged above 60.

Eight variables (mechanical ventilation, AKI, septic shock, creatinine level of > 1.13 mg/dL, urea > 7 mmol/L, ALT > 50 IU, AST > 50 IU, and platelet < 150 × 10^9^/L) were found to be associated with severe illness in this study. The independent predictor of severity included AKI, ALT > 50 IU and platelet < 150 × 10^9^/L. One of the main limitations was since the clinical data were collected only on the day of admission, we were not able to associate the time period and change in values for the predictors with the severe illness. A recent study from Malaysia also showed the association of ALT with severe leptospirosis [24]. In addition to the increased ALT, this previous study also reported abnormal lung sounds, hepatomegaly, hypotension, leukocytosis and low hematocrit as the predictors for severe leptospirosis. We found that low platelet level as one of the predictors of severe illness, however, this factor was not suggested in the above study. This present study and two other studies in Malaysia [13, 24] showed lung involvement as one of the predictors of severity and mortality in leptospirosis. Leptospirosis patients with pulmonary hemorrhage were also reported in several case reports including travelers who visited Malaysia [25–27]. These factors were found to be the independent factors for mortality in studies conducted in several other countries [8, 28, 29]. Similar to the present investigation, studies from other countries have also reported the association of exaggerated AST response and higher mean of aspartate/alanine aminotransferase ratio (AAR) with severity and mortality in leptospirosis [23]. These findings indicate that severe leptospirosis in Malaysia was characterized with multi-organs involvement. Progressive monitoring and measurements of these multi-factorial data even in patients presenting mild symptoms and with history of activities or exposure to potential source of *Leptospira* is vital to prevent the development of severe disease. Since these data were obtained during admission, patients are advised to seek medical treatment early if they presented with mild symptoms after jungle or water activities, exposed to flood or if working in areas with a high risk of rat infestation as the transition of mild to severe disease to fatality occur rapidly.

In our recent study, we identified two species of pathogenic and one species of intermediate *Leptospira (L. interrogans, L. kirschneri and L. wolffii)* causing leptospirosis in Central Malaysia with *L. interrogans* as the most common species [1]. The investigation on the association between infecting species and disease outcome (severe illness) did not show any significant association.

The data obtained from the present study provides baseline information on the factors associated with severe leptospirosis and this needs to be further evaluated on a larger sample size covering different geographical locations in Malaysia and across the globe.

## Conclusions

We identified mechanical ventilation, AKI, septic shock, creatinine level of > 1.13 mg/dL, urea > 7 mmol/L, ALT > 50 IU, AST > 50 IU, and platelet < 150 × 10^9^/L are associated with severe illness. AKI, high level of ALT and low level of platelet were defined as the independent factors for severity.

## Data Availability

The raw data/datasets used and/or analysed during the current study are available from the corresponding author upon request.

## Abbreviations

ARDS: Acute respiratory distress syndrome
SPHS: Severe pulmonary hemorrhage syndrome
IL-6: Interleukin 6
IL-17A: Interleukin-17A
IL-22: Interleukin-22
GIT: Gastrointestinal tract
HTAR: Hospital Tengku Ampuan Rahimah
PCR: Polymerase chain reaction
MAT: Microscopic agglutination test
WHO: World Health Organization
IMR: Institute for Medical Research
CI: Confidence interval
AKI: Kidney injury
ALT: Alanine aminotransferase
AST: Aspartate aminotransferase
HCT: Hematocrit
Hb: Hemoglobin
WBC: White blood cells
UN: Undefined
mg/dL: Milligrams per deciliter
mmol/L: Millimoles per liter
μmol/L: Micromoles per liter
IU: International unit
L: Liter
g/dL: Grams per deciliter
SD: Standard deviation
n: Total number
OR: Odds ratio
SE: Standard error
AAR: Aspartate/alanine aminotransferase ratio
